# Open Label Prospective Experience of Supplementation with a Fixed Combination of Magnesium, Vitamin B2, Feverfew, Andrographis Paniculata and Coenzyme Q10 for Episodic Migraine Prophylaxis

**DOI:** 10.3390/jcm10010067

**Published:** 2020-12-27

**Authors:** Michail Vikelis, Emmanouil V. Dermitzakis, George S. Vlachos, Panagiotis Soldatos, Konstantinos C. Spingos, Pantelis Litsardopoulos, Evangelia Kararizou, Andreas A. Argyriou

**Affiliations:** 1 Headache Clinic, Mediterraneo Hospital, 16675 Glyfada, Greece; gvlachos@neuromed.gr; 2 Glyfada Headache Clinic, 16675 Glyfada, Greece; 3 Headache Outpatient Clinic, 1st Department of Neurology, National and Kapodistrian University of Athens, 11528 Athens, Greece; ekarariz@med.uoa.gr; 4 Department of Neurology, “Geniki Kliniki” Euromedica, 54645 Thessaloniki, Greece; manolis.dermitzakis@gmail.com; 5 Private Practice, 24131 Kalamata, Greece; soldatosp@gmail.com; 6 Corfu Headache Clinic, 49131 Corfu, Greece; kcspigos@gmail.com; 7 Headache Outpatient Clinic, Neurology Department of the Saint Andrew’s State General Hospital of Patras, 26335 Patras, Greece; pantelis84@hotmail.com (P.L.); andargyriou@yahoo.gr (A.A.A.)

**Keywords:** episodic migraine, prevention, nutraceuticals, magnesium, vitamin B2, feverfew, coenzyme Q10

## Abstract

Background: To investigate the efficacy and safety of supplementation with a fixed combination of magnesium, vitamin B2, feverfew, andrographis paniculata and coenzyme Q10 in episodic migraine (EM) prevention. Methods: A pilot, single-arm, open-label study was conducted. After a one-month baseline period, the above-described supplementation was introduced in 113 EM Greek patients, who were prospectively followed-up for three months. The primary endpoint was the change in monthly migraine days between baseline period (BSL) and the third month of supplementation (T3). Secondary endpoints included changes in mean intensity of migraine and in days with use of acute migraine medications. Changes in scores of Migraine Disability Assessment questionnaire (MIDAS), Headache Impact Test-6 (HIT-6), Migraine Therapy Assessment questionnaire (MTAQ), Migraine-Specific Quality-of-life questionnaire (MSQ-QOL), Hospital Anxiety and Depression Scale (HADS) were also evaluated. Those with ≥50% reduction in monthly migraine days at T3, compared to BSL were considered supplementation-responders. Results: The mean number of migraine days was significantly decreased between BSL and T3 (9.4 ± 3.7 vs. 6.1 ± 3.5; *p* < 0.001). Likewise, days with peak headache intensity of >4/10 (5.7 ± 3.4 vs. 4.9 ± 3.1; *p* < 0.001) as well as days using acute headache medications per month (8.9 ± 3.6 vs. 5.7 ± 3.4; *p* < 0.001) were significantly reduced. At T3, 64 patients (56.6%) were classified as responders. The beneficial effect of supplementation was also associated with significant changes in HIT-6, MIDAS, MTAQ and MSQ-QOL scores. There were no safety concerns. Conclusions: The supplementation we have tested appears to be an effective and well-tolerated preventive approach against EM. A randomized, placebo-controlled study is needed to confirm our results.

## 1. Introduction

Migraine, a common primary headache disorder, ranks among the leading causes of all disease-associated disability worldwide and constitutes the major cause of disability among neurological disorders [1]. Based on its frequency, migraine can be classified as episodic (less than 15 days monthly) or chronic (more than 15 headache days monthly, of which at least 8 are of migrainous type or respond to migraine-specific medication, for more than 3 months) [2].

Apart from the use of the number of monthly migraine days to classify migraine in its episodic (EM) or chronic (CM) form, there are other phenotypic differences that can facilitate distinguishing the two conditions in order to establish a proper diagnosis. Generally, patients with EM have shorter average duration of headache, while they also experience less pain intensity, milder pain-associated autonomic symptoms, as well as pain-related comorbidities, compared to their counterparts with CM [3].

The course of both EM and CM over time, as well as the relationship between these conditions, are vaguely defined. There is evidence from large population-based studies that patients with EM can remit, remain stable, or even progress to CM at a rate of 2.5% per year, whereas the inverse can also occur, with an estimated 26% transition rate of CM to EM over a period of two years [4,5].

As such, apart from reducing migraine frequency, an additional critical goal of migraine prevention treatment is to hamper progression of EM to CM. So far there are no consensus guidelines clearly defining a specific phenotype in EM patients, which would likely benefit from prophylactic first-line therapies. Nonetheless, according to widely acknowledged guidelines, migraine prophylaxis should be considered when the frequency, intensity and duration of migraine attacks impose significant disability despite appropriate use of acute medications; when the frequency of migraine attacks and the excess use of acute medications make patients more liable to medication overuse headache (MOH), but also in patients with medical contraindications to acute migraine therapies. As already mentioned, an ultimate goal may be preventing progression of EM to CM [6].

Many conventional pharmacological medications are currently used in episodic migraine prophylaxis, including antihypertensives, antiepileptic drugs, beta and calcium channel blockers, and also various antidepressants. However, despite the fact that earlier studies indicate that treatment options with higher efficacy rates are preferred by patients even if side effects are present [7], more recent data clearly show that the use of the pharmacological preventive approaches is commonly associated with modest response, poor adherence and compliance [8] and a significant percentage of treatment discontinuations, estimated to be as high as 55% after 12 months of treatment [9]. 

The recent release of anti-*calcitonin gene-related peptide* (*CGRP*) monoclonal antibodies increased the expectations for a much higher efficacy/tolerability ratio in the prophylactic treatment of EM. Still, their wide use is limited by reimbursement policies across European countries, including Greece, where anti-CGRP compounds are not yet reimbursed and, therefore, a very limited national market occurs.

Nonetheless, since adverse events are the most common reason for early discontinuation of a migraine preventive treatment [9], it may be concluded that although efficacy may be high in patients’ preferences when starting a treatment, safety and tolerance probably play the most crucial role in the decision of stopping it early. In a more recent study from Greece, an astonishing 63% of participants claimed they would prefer the use of a neurostimulator for preventive treatment of migraine, over just 37% that preferred a pharmacological option [10], possibly reflecting the need for treatments that better balance efficacy and safety than traditional pharmacological treatments. Unfortunately, this study did not include the option of a nutraceutical treatment in the questionnaire used.

Generally, various nutraceuticals and nutritional supplements are widely utilized for migraine prophylaxis and may be a preferred option for patients with contraindications for pharmacological treatments, failure of previous treatments due to safety or tolerability or patients’ reluctance to use pharmacological treatments due to such concerns. A supplementation with a fixed combination of five nutraceutical agents, i.e., magnesium 281.25 mg, vitamin B 24.8 mg, feverfew150 mg, coenzyme Q10 20 mg, and andropraphis paniculata 100 mg is available in Greece and other European Union countries for migraine prevention (Vivinor^®^; Brain Therapeutics, Greece, also available in different European countries as Partena^®^; FB Health, Italy). In our study, we investigated the preventive ability of this supplementation in a population of Greek patients with episodic migraine.

## 2. Methods

### 2.1. Study Design

This open-label, single-arm, prospective, multicentre study was conducted in five headache outpatient centres located in five different nodal geographic locations of Greece, including the major urban areas of Athens, Thessaloniki, Patras, Kalamata and the island of Corfu. In accordance with the principles of the Helsinki Declaration as also with the Strengthening the Reporting of Observational studies in Epidemiology (STROBE) guidelines, eligibility was confirmed by a protocol-specific checklist and written informed consent was obtained from each patient. The study was approved by the principal investigator’s Institutional Review Board (Mediterraneo Hospital, Athens, protocol no 2718, 26 March 2018) and is registered at clinicaltrials.gov (NCT04463875).

### 2.2. Patient Selection

Participants were adult patients with a documented history of episodic migraine with or without aura for more than the 12 months prior to screening, according to the criteria of the International Classification of Headaches Disorders-III (IHS, 2018) [11]. The following inclusion criteria were applied: (1) established diagnosis of episodic migraine with or without aura for more than one year prior to study entry; (2) evidence of 4–14 migraine days per month during the last trimester prior to screening; (3) participants may had been either treatment-naive or not suitable for or had failed previous migraine pharmacological prophylactic treatments; (4) participants were able to fully understand protocol and study information provided by the investigators; and (5) enrolled patients should take no other preventive treatment or use any other migraine prophylactic method during the three months before entering the study and throughout the study period.

We excluded patients with the following criteria: (1) older than 50 years of age at migraine onset; (2) evidence of MOH; (3) pregnant or nursing females; (4) history of tension-type, cluster or hemiplegic headache; (5) history of severe anaphylactic reactions to any of the intervention’s ingredients; (6) evidence of severe systemic diseases; and (7) history or evidence of major psychiatric disorder.

### 2.3. Supplementation

After a one-month headache diary completion baseline phase, patients were started on three-month supplementation with one or two tablets daily, comprising of 281.25 mg magnesium, 4.8 mg vitamin B2, 20 mg coenzyme Q10, 150 mg feverfew and 100 mg andrographis paniculata, according to their treating physician’s decision and to standard clinical practice. The decision for use of one or two tablets was left at the treating physician’s discretion, according to his standard clinical practice and clinical judgment. The reasoning for use of two tablets would be to approach the standard preventive therapeutic dose of magnesium (600 mg), one the ingredients of the fixed supplementation for which there is the broadest experience, world-wide. On the other hand, the use of one tablet was justified by the hypothesis that a combination of agents with preventive action would provide a therapeutic result, despite the fact that the dose of the individual substances may be considered sub-therapeutical. The supplementation was given at a stable dose from onset through the end of study without up-titration. No deviation from the maximum target dose of two tablets per day was allowed. Demographic and clinical baseline data as well as response and safety profile of the supplementation were recorded and analyzed.

### 2.4. Assessments

At baseline visit (BSL) and after the informed consent procedure had been completed, physicians collected each patient’s demographic data, as well as migraine clinical phenotype characteristics, medical and migraine history, information on migraine attacks, associated symptoms and acute attack medications. A paper case report form (CRF) was used. V1 was followed by a one-month observation period (baseline phase, BSL). During BSL, patients were asked to complete a paper headache diary on a daily basis that included characteristics of the migraine phenotype, including number of days with migraine per month, headache intensity, associated symptoms, and use of acute medications.

At second visit (T2; Day 30 ± 10 since T1), patients experiencing between 4–14 migraine days during baseline period started supplementation. From T1 onwards until the end of the study at T3 (Day 120 ± 10), all patients kept the same paper headache diary in which they reported changes in the above-mentioned migraine characteristics longitudinally over time. Headache diary compliance was set at minimum of 80% of total days.

### 2.5. Efficacy Evaluation

The primary objective of our study was to evaluate the efficacy of the supplementation, as expressed by the change in mean number of migraine days between baseline period (BSL) and the third month of supplementation (T3). Secondary objectives included change in migraine severity between BSL and T3 as expressed by the change in the number of days with peak migraine intensity of more than 4 out of 10 in a 0–10 numerical scale (moderate/severe pain), and the change in days with any acute migraine medications used. Changes in scores of the Headache Impact Test-6 (HIT 6) [12]; Migraine Therapy Assessment questionnaire (MTAQ) [13]; MSQ (Migraine-Specific Quality of life) questionnaire [14] and of the Hospital Anxiety and Depression Scale (HADS) [15] were assessed between BSL vs. T3 as additional secondary endpoints. Change in *Migraine* Disability Assessment questionnaire (MIDAS) scores [16] between T2 and T3 was also a secondary endpoint.

Finally, data on patients’ preference and decision to continue treatment were also collected and analyzed. Patients with ≥50% (clinically significant) reduction in median migraine days during T3 compared to BSL were considered responders. Responders were further sub-classified as moderate responders (at least 50% reduction in migraine days); very good responders (at least 75% reduction in migraine days) and excellent responders (100% reduction in migraine days—migraine free).

### 2.6. Safety Evaluation

The current literature shows that supplementation with these specific active ingredients is generally safe and well tolerated [17] and as such no clinically-significant adverse events related to its use were expected to occur. Nonetheless, patients were encouraged to report any adverse events occurring throughout the study period either spontaneously or in response to general, indirect questioning. Each investigator was responsible for documenting the type and severity of overall adverse events and then categorized them for potential relationship to the supplementation given. Patients who received at least one dose of supplementation underwent safety evaluation.

### 2.7. Statistical Analysis

This was an exploratory study and as such no adjustment by multiplicity was made to account for the various endpoints considered. Sample size was determined in order to detect with a power of 80% and one-side 10% level of significance a 50% reduction in mean migraine days at the end of the study. To account for premature withdrawal of patients from the study, we increased the sample size by 5%.

Results were analyzed on an intention-to-treat basis. The primary intent-to-treat analysis included all enrolled patients (ITT population). A secondary efficacy analysis was performed on those patients who successfully completed the trial (EFF population), i.e., daily supplementation with a stable dose for 3 months. The primary efficacy variable (change in mean migraine days) was analyzed for both ITT and EFF populations. For the ITT analysis, early withdrawers for any reason, including perceived lack of efficacy, adverse events, intolerance or other were counted as non-responders per se.

Descriptive data analysis included categorical variables presented in counts and weighted percentages, and continuous variables as mean or median with the corresponding standard error or range, depending on the nature of the variable. The changes in mean clinical scores from BSL vs. T3 were assessed using paired samples t-tests, after checking whether the variables followed the normal distribution with the Kolmogorov–Smirnov test. The Χ^2^ test was used to ascertain differences between categorical variables. Estimates of effect size were computed using the Cohen’s *d* for paired samples *t*-tests. Correlations between baseline demographic and other neurological characteristic of patients and the rate of responders (≥50%) to supplementation were examined using the Spearman’s rank correlation coefficient. All tests were two-sided, unless otherwise stated, i.e., sample size power determination which was one-sided. Calculations were performed using SPSS software package version 23.0 (SPSS Inc., Chicago, IL, USA). *p* values of <0.05 were considered significant.

## 3. Results

We enrolled a total of 113 patients, 22 males and 93 females, with a mean age of 39.1 ± 12.4 years. Among female participants, 24/93 (25.8%) were in menopausal status. All of them successfully completed the study, without any event of early withdrawal. Hence, both the ITT and EFF populations were comprised of the same sample size (*n* = 113 patients). Among them, 54 (47.8%) were preventive treatment-naïve for their migraine, whereas 59 (52.2%) patients had failed in a mean number of 2.3 ± 1.1 (range: 1–5) previous medications, like flunarizine, valproic acid, topiramate, propranolol and amitriptyline. Their demographic and baseline clinical characteristics are summarized in Table 1.

**Figure 1:**
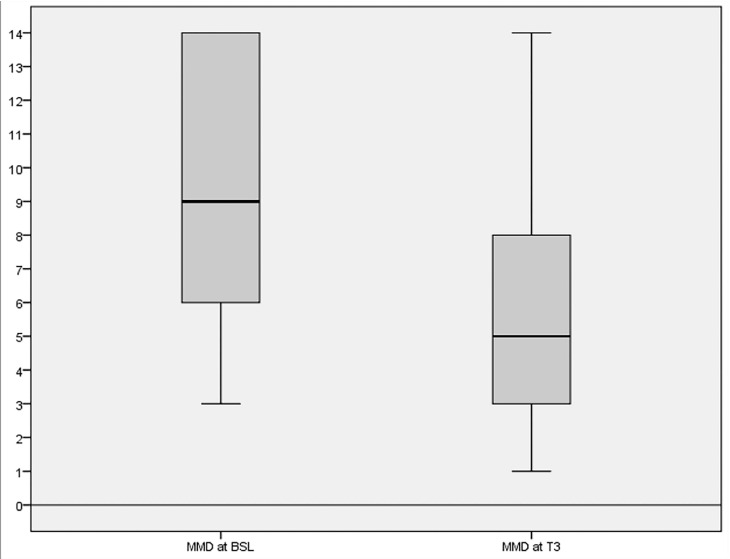
Changes in mean migraine days (MMD) from baseline (BSL) to the third month of supplementation (T3).

The analysis of the primary response variable (*n* = 113) showed that there was a statistically significant decrease in mean migraine days (9.4 ± 3.7 vs. 6.1 ± 3.5; *p* < 0.001-Cohen’s *d*: 1.145) between BSL and T3 (Figure 1). This effect remained significant both for males (*n* = 22; 8.9 ± 4.0 vs. 4.8 ± 2.7; *p* < 0.001) and females (*n* = 93; 9.4 ± 3.7 vs. 6.3 ± 3.6; *p* < 0.001). Moreover, migraine severity was also significantly decreased as measured by the change in the number of monthly days with peak migraine intensity of more than four (moderate/severe pain) on a 0–10 numerical scale between BSL and T3 (5.7 ± 3.4 vs. 4.9±3.1; *p* < 0.001-Cohen’s *d*: 0.984). Supplementation was also associated with a significant reduction in days using acute migraine medications per month between BSL and T3 (8.9 ± 3.6 vs. 5.7 ± 3.4; *p* < 0.001-Cohen’s *d*: 1.021).

The beneficial effect of active supplementation was evident in both EM patients with aura (*n* = 24/113; 21.2%) and without aura (*n* = 89/113; 78.8%). The mean migraine days significantly decreased between BSL and T3 in patients with aura (10.1 ± 3.7 vs. 6.7 ± 3.5; *p* < 0.001), but also in those without aura (9.0 ± 3.7 vs. 5.9 ± 3.3; *p* < 0.001).

At T3, 64 patients (56.6%) had experienced a ≥ 50% reduction in mean migraine days during the third month of supplementation compared to BSL and were therefore classified as responders. Among all (*n* = 64) responders, 59 achieved response at 50% (52.2%) and 5 at 75% (4.4%). The remaining 49 patients (43.4%) reported less than 50% reduction with supplementation. It should be noted that seven of them (6.2% of total study population) achieved a moderate 30–49% reduction in migraine days. Among the remaining non-responders, 22/42 (19.5%) experienced no benefit at all, while 20/42 (17.7% of total study population) patients achieved less than a 30% reduction in mean migraine days.

The rate of responders at ≥50% to supplementation at T3 remained unrelated to age, gender or any other baseline demographic or other neurological characteristic of patients, including supplementation dosage with either one (n = 26; 23%) or two tablets (*n* = 87; 77%) per day at maintenance (Spearman’s rho; *p* = 0.570), as also evidence of failure in previous preventive medications (Spearman’s rho; *p* = 0.594).

As can be seen in Table 2, the beneficial effect of active supplementation was also associated with statistically significant changes in secondary endpoints, i.e., HIT 6, MTAQ and MSQ-QOL scores between BSL and T3 and in MIDAS scores, between T2 and T3. In contrast, HADS scores remained unchanged over time. The clinically significant improvement in migraine frequency and severity at T3 compared to BSL was strongly associated with better QOL outcomes (*p* < 0.001) according to MSQ-QOL questionnaire scores. Notably, a total of 70 participants remained satisfied from the study intervention and all of them continued further supplementation after the completion of the study. This effect remained unrelated to supplementation dosage of either one or two tablets.

Active supplementation also proved to be safe and well tolerated. In total, five patients reported diarrhea, which was mild in all cases and patients were able to complete the study with some temporary adjustment in their diet. No other adverse events were reported.

## 4. Discussion

The pathophysiology of migraine likely involves dysfunction of subcortical structures modulating sensory input from the trigeminovascular system. As a result, vasoactive peptides, such as *CGRP* and substance P, are released from trigeminovascular neurons, thereby exacerbating vasodilation and generating neurogenic inflammation [18,19]. Mitochondrial dysfunction, increased calcitonin, matrix metalloproteinase 9 (MMP-9), and nitric oxide (NO) levels, as well as decreased level of metabolic enzymes are also considered among the significant factors generating migraine [20]. Additionally, genetic and environmental factors might also be involved in triggering the onset of migraine attacks [21].

Various conventional pharmacological treatments for migraine prevention are currently in use, aiming to reduce afferent traffic or stabilizing these above-mentioned abnormal pathways [18]. Nonetheless, many patients respond poorly to, or experience adverse events with these treatments [8]. In addition, many patients are noncompliant with these medications; unsatisfactory efficacy, safety or tolerability issues, and concerns about long-term safety are among the reasons [9].

As such, there has been a growing therapeutic shift over the last few years towards treatments with lower adverse event rate, including onabotulinum toxin-A, monoclonal antibodies, external neurostimulators [22,23,24] and nutraceuticals. Nutraceuticals is a non-pharmacological approach that includes vitamins, minerals, and herbs in the prevention of migraines. The level of evidence to support use of nutrients is low or moderate, mainly because of lack of rigorous clinical trials. Nonetheless, patients often prefer nutraceutical treatment over traditional pharmacological approaches in migraine prophylaxis to diminish possible side effects and intolerance, but also based on the belief that herbal remedies or nutrients are much safer than drugs [25,26].

The use of nutraceuticals is included or accepted by various guidelines despite the rather poor or moderate level of evidence, in both the EU and US [6,17], based on the lack of significant adverse events and the potential of an individual or synergistic ability to target significant factors involved in migraine pathogenesis [25]. Interestingly, the Canadian Headache Society Guideline for Migraine Prophylaxis [27] includes riboflavin, coenzyme Q10, and magnesium citrate in the list of prophylactic drugs that received a strong recommendation for use, along with topiramate, propranolol and amitriptyline, among others. This recommendation comes despite the rather poor or moderate level of evidence, as authors of the Canadian Headache Society Guideline for Migraine Prophylaxis acknowledge, and is mainly based on the safety and tolerability profile, an approach which seems rational and reflecting the real-world situation.

Indeed, existing knowledge shows that magnesium, vitamin B2, feverfew and coenzyme Q10 are helpful in migraine prophylaxis with minimal safety issues, as these nutrients might be able to target some of the processes involved in migraine pathogenesis [28]. Specifically, magnesium blocks glutamate receptors, modulates ATP production and glucose metabolism and as such high dose supplementation is able to decrease glutamate-activated cortical spreading depression. Likewise, high dose supplementation with vitamin B2 and coenzyme Q10 may augment activity of mitochondrial complexes to prevent mitochondrial dysfunction. Finally, the use of feverfew is attributed to its properties to inhibit serotonin release from platelets and evoke vascular smooth muscle relaxation [25,29].

In the current setting, we documented a significant improvement in all primary and secondary efficacy variables (excepting HADS-A and HADS-D) after 3 months of supplementation with a proprietary fixed combination of magnesium, vitamin B2, feverfew, coenzyme Q10 and androgrpahis paniculata. A total of 64/113 (56.6%) enrolled EM patients with or without aura obtained a response rate at ≥50%, which was associated with improved HIT-6, MIDAS, and MSQ-QOL scores (*p* < 0.001). An even larger group of patients (*n* = 70; 62%) remained satisfied from treatment and wished to continue supplementation, thoroughly bolstering the view that some migraineurs prefer nutraceutical over pharmacological approaches in order to avoid side effects even if the response is less clinically significant, i.e., at 30%. Notably, more than half of our patients had tried up to five previous prophylactic pharmacological medications before being supplemented and either experienced modest efficacy or poor tolerance due to side effects. As such, based on their experience, they preferred to use a potentially less effective but considered safe complementary medication as monotherapy [10,25].

Patient satisfaction and a decision to continue a specific preventive migraine treatment is important in clinical practice and may not always be completely related to outcomes usually used in clinical trials. In other words, patients experiencing modest improvement, less than the 50%, may be satisfied and willing to continue or repeat a treatment if they experience little or no side effects and are confident that severe side-effects are unlikely in the future. On the other hand, a patient experiencing significant improvement may be reluctant to continue or repeat a treatment because of concerns with current or potential side effects or long-term safety.

Finally, another main observation of the current study is that none of the enrolled patients withdraw her/his participation in the study and discontinued supplementation early throughout the process due to intolerable adverse events or due to lack of efficacy, despite the fact that some of the participants unarguably did not benefit from this treatment. Our results, overall, are in agreement with a previously published study applying a similar study design to test a proprietary supplement containing feverfew, magnesium and Q10 [30]. Improvement of migraine symptoms with a proprietary supplement containing riboflavin, magnesium and Q10 was also noted in a randomized, placebo-controlled, double-blind, multicenter trial enrolling 130 migraineurs, although reduction in migraine frequency showed only a trend towards statistical significance between active and placebo arms [31]. However, direct comparison between the results of the latter study and ours is difficult because of methodological differences.

Different dosages and formulations as well methodological issues in the study design (observational open-label vs. placebo-controlled study design) may account for the discrepancy between results of the latter trials. In any case, despite that several studies and reviews on nutraceuticals have already evaluated the same active substances of Vivinor^®^ (despite at different dosage) individually, we tested and report for the first time the efficacy/tolerability ratio of a fixed combination of magnesium, vitamin B2, feverfew, andrographis paniculata and coenzyme Q10 in EM prevention; thereby adding new knowledge in the existing body of evidence. One could argue that this combination itself might impede the identification of the “culprit” that may induce a possible beneficial clinical effect on migraine as many of the compounds contained in the combination we have herein tested (one of the many on the market), taken alone were found to be effective in migraine prophylaxis, but at a higher dosage. For instance, coenzyme Q10 was effective in a double-blind study at a dosage of 300 mg/day [32] and Vit B2 at a dosage of 400 mg [33].

In any case, our experience with this fixed combination showed that this supplementation may be an effective and well tolerated complementary treatment in EM prophylaxis. However, the pilot open-label design of this trial, the lack of a control group and the potential for selection or response bias, also present in other similar studies, can be acknowledged as significant limitations. In particular, the open-label design of our study and the absence of a control group with placebo or active treatment does not leave room for firm conclusions. Still, our study is a pilot one and a well-designed, blinded, controlled study should follow, in order to reach certainty. It also should be taken into account that the product is currently available in several European countries, while according to regulatory authorities, nutraceuticals are not required to have registered double-blind controlled studies prior to their release [34,35].

The adoption of two different supplemenation dosages (one or two tablets) can also be perceived among the limitations of our study, although the beneficial effect of our supplementation remained unrelated to this dichotomy, in agreement with the comparable efficacy of 3 months exposure to either low (70 mg) or high dose erenumab (140 mg) in the treatment of episodic and chronic migraine [36,37].

Nevertheless, and to best possibly support the positive outcomes of this specific supplementation, we used migraine-specific tools as endpoints to assess changes in disability, psychological burden, QOL, and satisfaction in close relation to the intervention we tested.

## 5. Conclusions

Further larger placebo-controlled trials are warranted to confirm our results on the potential beneficial effect for this proprietary supplement, containing magnesium, vitamin B2, feverfew, coenzyme Q10 and andrographis paniculata in EM prophylaxis.

All participants have given signed consent for publication of the present material.

## Figures and Tables

**Table 1:** Patients’ baseline and clinical characteristics.

Variable	Study Sample N = 113 N (%)
**Sex**	
Males	20 (17.7)
Females	93 (82.3)
**Age ± SD**	39.1 ± 12.4
**Height in cm ± SD**	168.4 ± 7.8
**Weight in kgr ± SD**	64.5 ± 10.7
**Age at migraine onset**	
10–18 years	44 (38.9)
18–25 years	43 (38.1)
25–30 years	16 (14.2)
30–40 years	8 (7.1)
40–50 years	2 (1.8)
**Migraine type**	
Aura	24 (21.2)
Non-Aura	89 (78.8)
**Supplementation (tablet) dose**	
1 tablet/day	26 (23.0)
2 tablets/day	87 (77.0)

**Table 2:** Changes in outcome measures assessing secondary efficacy variables from baseline (BSL) to the last month of trimester (Τ3) of supplementation with in 113 patients comprising both the efficacy and intention to treat population.

Tools Assessing Secondary Endpoints	BSL	T3	*p* Value
	Mean ± SD Median	Mean ± SD Median	
**HIT-6**	68.6 ± 5.7 69	63.8 ± 10.3 63	*p* < 0.001
**MIDAS**	67.0 ± 43.8 58	49.5 ± 41.0 30	*p* < 0.001
**MTAQ**	2.9 ± 1.1 3	3.2 ± 0.9 4	*p* < 0.001
**MSQ-QOL total**	52.3 ± 15.4 52	71.3 ± 10.4 71	*p* < 0.001
**HADS-A**	8.3 ± 5.8 6	8.3 ± 8.5 7	*p* = 0.923
**HADS-D**	5.7 ± 3.7 5	6.1 ± 5.2 4	*p* = 0.216

Abbreviations: Headache Impact Test-6 (HIT 6); *Migraine* Disability Assessment questionnaire (MIDAS); Migraine Therapy Assessment questionnaire (MTAQ); Migraine-Specific Quality of life questionnaire (MSQ-QOL) and Hospital Anxiety and Depression Scale (HADS) anxiety (A) and depression (D).

## Data Availability

All data and materials are available upon request.

## Reporting Checklist

We present the following article in accordance with the STROBE reporting checklist.
